# Role of spinal cord glutamate transporter during normal sensory transmission and pathological pain states

**DOI:** 10.1186/1744-8069-1-30

**Published:** 2005-10-21

**Authors:** Yuan-Xiang Tao, Jianguo Gu, Robert L Stephens

**Affiliations:** 1 Department of Anesthesiology and Critical Care Medicine, Johns Hopkins University School of Medicine, 355 Ross, 720 Rutland Ave., Baltimore, Maryland 21205, USA; 2 Department of Oral and Maxillofacial Surgery, Mcknight Brain Institute and College of Dentistry, University of Florida, Gainesville, Florida, 32610, USA; 3 Department of Physiology and Cell Biology, The Ohio State University College of Medicine, Columbus, Ohio 43210, USA

## Abstract

Glutamate is a neurotransmitter critical for spinal excitatory synaptic transmission and for generation and maintenance of spinal states of pain hypersensitivity via activation of glutamate receptors. Understanding the regulation of synaptically and non-synaptically released glutamate associated with pathological pain is important in exploring novel molecular mechanisms and developing therapeutic strategies of pathological pain. The glutamate transporter system is the primary mechanism for the inactivation of synaptically released glutamate and the maintenance of glutamate homeostasis. Recent studies demonstrated that spinal glutamate transporter inhibition relieved pathological pain, suggesting that the spinal glutamate transporter might serve as a therapeutic target for treatment of pathological pain. However, the exact function of glutamate transporter in pathological pain is not completely understood. This report will review the evidence for the role of the spinal glutamate transporter during normal sensory transmission and pathological pain conditions and discuss potential mechanisms by which spinal glutamate transporter is involved in pathological pain.

## 

In addition to its essential metabolic role, glutamate is a major mediator of excitatory signals in the central nervous system and is involved in many physiologic and pathologic processes, such as excitatory synaptic transmission, synaptic plasticity, cell death, stroke, and chronic pain [1,2]. Glutamate exerts its signaling role by acting on glutamate receptors, including *N*-methyl-D-aspartate (NMDA), α-amino-3-hydroxy-5-methyl-4-isoxazolepropionic acid (AMPA)/kainate, and metabotropic glutamate receptors. These receptors are located on the pre- and post-synaptic membranes, as well as, at extra-synaptic sites. Glutamate concentration in the synaptic cleft determines the extents of receptor stimulation and excitatory synaptic transmission. It is of critical importance that the extracellular glutamate concentration be kept at physiological levels, as excessive activation of glutamate receptors can lead to excitotoxicity and neuronal death [3]. The clearance of glutamate from the synaptic cleft is principally dependent on Na^+^-dependent, high-affinity, neuronal glutamate transporters present presynaptically, postsynaptically, and perisynaptically, and on glial glutamate transporters (Fig. 1). Currently, five isoforms of glutamate transporters have been identified [3]: namely, GLAST (glutamate/aspartate transporter), GLT-1 (glutamate transporter-1), EAAC (excitatory amino acid carrier) 1, EAAT (excitatory amino-acid transporter) 4, and EAAT5. The human homologues of the three more ubiquitous subtypes (GLAST, GLT-1, and EAAC1) are named EAAT1, EAAT2, and EAAT3, respectively. The five isoforms belong to the same gene-family and share 50–60% amino acid sequence identity [3]. However, they have discrete cellular and regional localizations. GLAST is present in glial cells throughout the central nervous system, with strong labeling in cerebellar Bergmann glia and more diffuse labeling in the forebrain [3]. It is also transiently expressed in a small number of neurons [4]. GLT-1 is almost exclusively expressed on glia and is widespread and abundant throughout the forebrain, cerebellum, and spinal cord [4]. In contrast, EAAC1 is found predominantly in neurons of the spinal cord and brain [4,5]. EAAT4 has properties of a ligand-gated Cl-channel and is localized mainly in cerebellar Purkinje cells [6]. EAAT5 is retina-specific [7].

**Figure 1:**
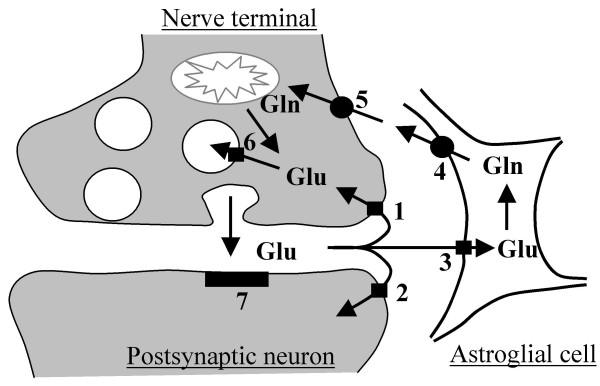
Glutamate (Glu) uptake and Glu/glutamine (Gln) cycle. Glu released from the nerve terminal by exocytosis is taken up by neuronal Glu transporter present presynaptically (1) and postsynaptically (2) and by glial Glu transporter (3). Glu/Gln cycle is one type of Glu recycling, but the significance is still unclear *in vivo *(see references 37 and 38). Astroglia detoxifies Glu by converting it to Gln. Glu is subsequently released from the glial cells by glial Gln transporter (4) and taken up by neuronal Gln transporter (5). Neurons convert Gln back to Glu, which is loaded into synaptic vesicles by vesicular Glu transporter (6). 7: postsynaptic Glu receptors.

Given the well-documented evidence that glutamate acts as a major excitatory neurotransmitter in primary afferent terminals [2], it is expected that glutamate transporter might be involved in excitatory sensory transmission and pathological pain. Indeed, recent studies have revealed that inhibition of spinal glutamate transporter produced pro-nociceptive effects under normal conditions [8] and have unexpected antinociceptive effects under pathological pain conditions [9-11]. It is not completely understood why the effects of spinal glutamate transporter inhibition under pathological pain conditions are opposite to its effects under normal conditions. In this review, we will illustrate the expression and distribution of the glutamate transporter in two major pain-related regions: spinal cord and dorsal root ganglion (DRG). We will also review the evidence for the role of the glutamate transporter during normal sensory transmission and pathological pain conditions and discuss potential mechanisms by which glutamate transporter is involved in pathological pain.

## Expression and distribution of glutamate transporter in the spinal cord and dorsal root ganglion

**Figure 2:**
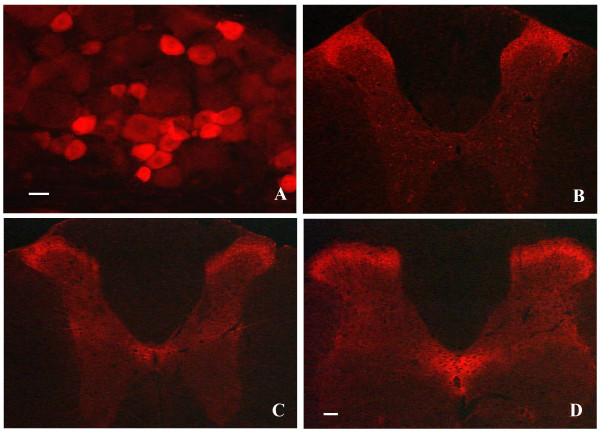
Expression and distribution of the glutamate transporter in the dorsal root ganglion (A) and the spinal cord (B-D). EAAC1 is expressed mainly in small dorsal root ganglion cells (A) and distributed predominantly in the superficial dorsal horn of the spinal cord (B). GLAST (C) and GLT-1 (D) are expressed highly in the superficial dorsal horn and the region around the central canal. Scale bars: 10 μm in A and 125 μm in B, C, and D.

**Figure 3:**
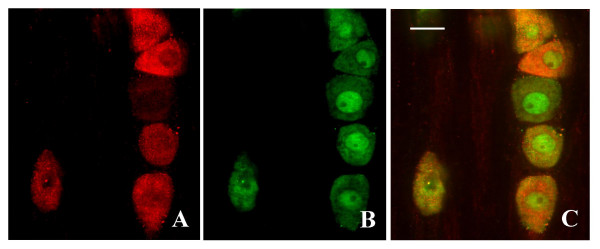
Double-immunofluorescence histochemistry for EAAC1 (A) and NeuN (B, a marker for neuronal nuclei), and their overlapping (C) in the dorsal root ganglion. Scale bar: 10 μm.

In the spinal cord, three isoforms of glutamate transporter (GLAST, GLT-1, and EAAC1) have been reported [4,12]. They are expressed in highest density within the superficial dorsal horn of the spinal cords of rats and mice (Fig. 2). GLT-1 and GLAST are exclusively distributed in glial cells at perisynaptic sites in the superficial dorsal horn [13]. EAAC1, in addition to its expression in the spinal cord neurons, is detected in the DRG and distributed predominantly in the small DRG neurons (but not in DRG glial cells) [12] (Fig. 3). Some of these EAAC1-positive DRG neurons are positive for calcitonin gene-related peptide (CGRP) or are labeled by IB4 [12,13]. Unilateral dorsal root rhizotomy shows less intense EAAC1 immunoreactivity in the superficial dorsal horn on the ipsilateral side, compared to the contralateral side [12]. Moreover, confocal microscopy demonstrates that some EAAC1-positive, small dot- or patch-like structures in the superficial laminae are labeled by IB4 or are positive for CGRP [12]. Under electron microscope, EAAC1 is associated with the axon terminal and dendritic membranes at synaptic and non-synaptic sites and is present with CGRP in the axons and the terminals in the superficial dorsal horn [13]. The expression level and distribution pattern of neuronal and glial glutamate transporters in the superficial dorsal horn suggest an important role for spinal glutamate transporter in spinal nociceptive transmission. In addition, the unique expression of EAAC1 in the small DRG neurons and nociceptive primary afferent terminals suggests that EAAC1 might have a distinct role in pain processing, compared to GLT-1 and GLAST.

## Role of the spinal cord glutamate transporter in normal sensory transmission

Recently evidence suggests that spinal glutamate transporter might play an important role in normal sensory transmission. Liaw et al. [8] reported that intrathecal injection of glutamate transporter blockers DL-threo-β-benzyloxyaspartate (TBOA) and dihydrokainate (DHK) produced significant and dose-dependent spontaneous nociceptive behaviors, such as licking, shaking, and caudally directed biting, phenomena similar to the behaviors caused by intrathecal glutamate receptor agonists, such as glutamate, NMDA, or AMPA, when given intrathecally [14-16]. Intrathecal TBOA also led to remarkable hypersensitivity in response to thermal and mechanical stimuli [8]. These findings are consistent with a previous report that showed an increase in spontaneous activity and responses of wide dynamic range neurons to both innocuous mechanical (brush, pressure) and noxious mechanical (pinch) stimuli after topical application of L-trans-pyrrolidine-2,4-dicarboxylic acid (PDC), a glutamate transporter blocker [17,18]. TBOA-induced behavioral responses could be significantly blocked by intrathecal injection of the NMDA receptor antagonists MK-801 and AP-5, the non-NMDA receptor antagonist CNQX or the nitric oxide synthase inhibitor L-NAME [8]. The effects of DHK and PDC were thought to be partially due to their non-specific interactions with glutamate receptors. However, unlike DHK and PDC, TBOA does not act as an agonist or antagonist at glutamate receptors [9,19,20]. Thus, spontaneous pain-related behaviors and sensory hypersensitivity evoked by TBOA directly support the involvement of glutamate transporter in normal excitatory synaptic transmission in the spinal cord. *In vivo *microdialysis analysis showed that intrathecal injection of TBOA produced short-term elevation of extracellular glutamate concentration in the spinal cord [8]. Topical application of TBOA on the dorsal surface of the spinal cord also resulted in a significant elevation of extracellular glutamate concentrations demonstrated by *in vivo *glutamate voltametry [8]. These findings indicate that a decrease of spinal glutamate uptake can lead to excessive glutamate accumulation in the spinal cord, which might, in turn, result in over-activation of glutamate receptors, and production of spontaneous nociceptive behaviors and sensory hypersensitivity. Thus, glutamate uptake through spinal glutamate transporters is critical for maintaining normal sensory transmission under physiological conditions.

## Expression and function of the spinal cord glutamate transporter in pathological pain states

Glutamate uptake and expression of glutamate transporters in the spinal cord have been found to be changed under pathological conditions associated with chronic pain status. Chronic constriction nerve injury upregulated glutamate transporter expression at day 1 and 4 postoperatively, but it downregulated glutamate transporter expression at days 7 and 14 postoperatively [21]. Moreover, chronic constriction nerve injury significantly reduced spinal glutamate uptake activity at day 5 postoperatively [21]. Recently, another study showed that spinal nerve ligation also markedly reduced glutamate uptake activity, as demonstrated in spinal deep dorsal and ventral horn 4–6 weeks after the nerve ligation [22]. Although the underlying mechanism by which neuropathic inputs cause the decrease in spinal glutamate uptake is unclear, it is thought that this decrease might contribute to the central mechanisms of the development and maintenance of pathological pain[21,22].

As shown above, inhibition of glutamate uptake produces pronociceptive effects in normal animals [8]. Unexpectedly, in pathological pain states, inhibition of glutamate transporter activity produced antinociceptive effects. For example, glutamate transporter inhibitors attenuated the induction of allodynia induced by PGE_2_, PGF_2α_, and NMDA [9]. Inhibition or transient knockdown of spinal GLT-1 led to a significant reduction of nociceptive behavior in the formalin model [10]. Consistent with these findings, the preliminary work from Yuan-Xiang Tao's laboratory showed that three different glutamate transporter inhibitors (TBOA, DHK, threo-3-hydroxyaspartate) reduced formalin-induced nociceptive responses and Complete Freund's adjuvant (CFA)-evoked thermal hyperalgesia [11]. On the other hand, the glutamate transporter activator MS-153, which is reported to accelerate glutamate uptake in *in vivo *and *in vitro *studies [23-26], had no effect in formalin tests when MS-153 was applied via intrathecal injection, even at the highest dose (1,000 μg/10 μl) [11]. Interestingly, Sung et al. reported that riluzole, a glutamate transporter regulator, significantly attenuated thermal hyperalgesia and mechanical allodynia after chronic constriction nerve injury [21], but this drug was ineffective against peripheral neuropathic pain in a clinical setting [27]. The reason for the discrepancy between the two studies is unclear, but it is worth noting that, in addition to increasing glutamate uptake, riluzole has multiple actions on many systems [neuroprotective, anticonvulsant, anxiolytic, and anesthetic qualities by its blockade of sodium channel α-subunits, glutamate receptors, and γ-aminobutyric acid (GABA) reuptake and its stabilization of voltage-gated ion channels] [28-31]. Thus, more selective drugs that promote spinal glutamate transporter function are needed to demonstrate whether glutamate transporter activators have possible efficacy in the treatment of chronic pain.

**Figure 4:**
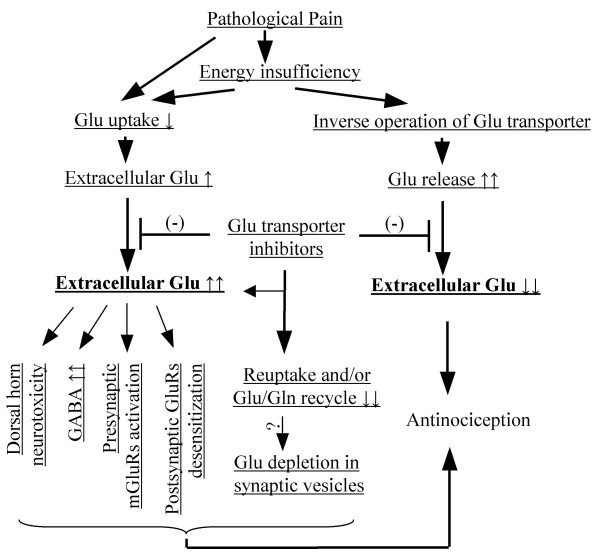
Two distinct models for the role of glutamate (Glu) transporter inhibitors in pathological pain. Pathological pain might cause Glu uptake decrease and energy insufficiency in the spinal cord. The latter may, in turn, results in Glu uptake decrease as well as inverse operation of spinal Glu transporter to release Glu. In one model, Glu transporter inhibitors further block Glu uptake and enhance the increase in extracellular Glu levels, perhaps leading to dorsal horn neuronal death, increase of spinal GABA contents, activation of inhibitory presynaptic mGluRs, and desensitization of postsynaptic Glu receptors. Glu transporter inhibitors also block neuronal Glu transporter reuptake and/or the Glu/Gln cycle via inhibition of glial Glu transporter, resulting in Glu depletion of synaptic vesicles in primary afferent terminals. In contrast, Glu transporter inhibitors block inverse operation of Glu transporter to release Glu and decrease extracellular Glu levels in another model.

The intriguing question remains as to why glutamate transporter inhibitors have antinociceptive effects under pathologic pain conditions that are opposite to their pro-nociceptive effects under normal conditions. Several mechanisms potentially contribute to the role of the glutamate transporter inhibitors under pathological pain states (Fig. 4). First, the blockade of spinal glutamate transporter uptake inhibits clearance of glutamate, leading to the chronic elevation of spinal extracellular glutamate, possibly subsequently causing excitotoxicity, compromising or destroying subsceptible dorsal horn neurons, and interfering with the transmission of pain signaling. However, preliminary data from Dr. Tao's laboratory showed that transient glutamate transporter inhibition did not produce significant spinal neuronal damage in rat formalin or CFA model [11]. Second, GABA, an inhibitory transmitter, is synthesized from glutamate by glutamic acid decarboxylase. Do the increased glutamate levels caused by glutamate transporter inhibition lead to increased GABA in the spinal cord? Recent work showed that inhibition of glutamate transporter activity depleted both glutamate and GABA neurotransmitter pools and reduced inhibitory postsynaptic current (IPSC) and miniature IPSC amplitudes [32,33]. Thus, if this property extends to the spinal cord, one would expect that blockade of spinal glutamate transporter would decrease the amount of GABA in GABAergic terminals and reduce IPSP or IPSC. This expectation would not explain the mechanisms of antinociception by glutamate transporter blocker in pathological pain. Third, inhibition of reuptake through presynaptic EAAC1 and/or the glutamate/glutamine cycle in the spinal cord might result in a depletion of glutamate in synaptic vesicles and a decrease in presynaptically released glutamate, leading to a reduction in glutamate receptor-mediated nociceptive transmission. It is documented that abundant glutamate is distributed in intracellular space, particularly inside nerve terminals [3,34,35]. As a precursor for transmitter glutamate, glutamine is also rich in the intracellular and extracellular fluid [3]. Moreover, although it has been demonstrated *in vitro *that glutamine is a precursor of transmitter glutamate, the *in vivo *evidence regarding the glutamate/glutamine cycle is less convincing [36]. The ability of glutamatergic neurons to sustain release of glutamate independently of glutamine might be related to a newly found capacity for pyruvate carboxylation [37,38]. Pyruvate carboxylation replenishes the loss of α-ketoglutarare from the tricarboxylic acid cycle that is inherent in release of glutamate. Thus, inhibition of spinal glutamate transporter might not cause the depletion of glutamate in presynaptic vesicles in pathological pain. Fourth, chronic elevation of extracellular glutamate caused by glutamate uptake inhibition might activate the inhibitory presynaptic metabotropic glutamate receptors (mGluRs) [39-41] and promote a postsynaptic desensitization of glutamate receptors [40]. It is possible that, during pathological pain conditions, glutamate transporter inhibitor-produced antinociception might be due to the decreased release of pre-synaptic glutamate via activation of inhibitory mGluRs in primary afferent terminals and/or reduced postsynaptic efficacy of glutamate via desensitization of glutamate receptors in the spinal dorsal horn neurons. Finally, glutamate transporter inhibitors might produce antinociception in pathological pain by the mechanism of blocking inverse operation of the glutamate transporter. It is well documented that the glutamate transporter imports one glutamate ion and co-transports three Na^+ ^ions into the cell [42] and that transporter function is dependent upon both the membrane potential and the transmembrane ion gradients established by Na^+^-K^+^ATPase as driving forces [43,44]. Under physiological conditions, these forces are sufficient to maintain the concentration gradient of micromolar extracellular glutamate against millimolar intracellular glutamate through glutamate transporter uptake [3,42]. However, under pathological conditions, metabolic insults that deplete intracellular energy and alter ionic gradients can lead to reversed action of the glutamate transporter [3]. For example, during brain ischemia, ATP is depleted and impairment of Na^+^-K^+^ATPase results in the increases in intracellular Na^+ ^ions and extracellular K^+ ^ions, which causes inverse operation of the glutamate transporter and release of glutamate into the extracellular space [3]. Indeed, the glutamate transporter inhibitors (e.g., TBOA) reduce glutamate release and have neuroprotective actions in brain ischemia [20]. Does pathological persistent pain cause cellular energy insufficiency that inverses the glutamate transporter operation to release glutamate in the spinal cord? Metabolic activity and energy demand significantly increase in the spinal cord under pathological pain conditions [45-48]. Such hyperactive states of spinal neuronal and glial cell activities might not only consume large amounts of cell energy, but also disturb energy metabolism, decrease ATP, and result in energy insufficiency that might reverse spinal glutamate transporter operation to release glutamate. It is possible that blocking the reversed glutamate transporter-mediated glutamate release is an underlying mechanism of antinociception produced by glutamate transporter inhibition under chronic pain conditions.

Taken together, it is evident that at least five potential mechanisms are involved in the action of glutamate transporter inhibitors during pathological pain (Fig. 4). In the first four mechanisms, glutamate transporter inhibitors lead to an increase in spinal extracellular glutamate levels, whereas, in the last one, glutamate transporter inhibitors block the reversed glutamate transporter-mediated glutamate release, and reduce extracellular glutamate levels (Fig. 4). Therefore, two distinct models explain the role of spinal glutamate transporter in pathological pain (Fig. 4). Determining extracellular glutamate levels in the spinal cord following glutamate transporter inhibition during pathological pain might be a key to determine the mechanisms of glutamate transporter inhibitor-produced antinociception in the state of pathological pain.

## Conclusion

Pathological pain, particularly as a result of nerve injury, is poorly managed by current drugs, such as opioids and non-steroidal anti-inflammatory drugs. Glutamate receptor antagonists are effective in reducing pain hypersensitivity in animal models and clinical settings, but with unacceptable side effects. Glutamate transporter inhibitors have recently been shown to produce antinociceptive effects in several preclinical pathological pain models. Further studies to delineate the role of the spinal glutamate transporters during chronic pain states might lead to better strategies for the prevention and therapy of chronic pain.
